# Metabolic analysis of sarcopenic muscle identifies positive modulators of longevity and healthspan in *C. elegans*

**DOI:** 10.1016/j.redox.2025.103732

**Published:** 2025-06-14

**Authors:** Steffi M. Jonk, Alan Nicol, Vicki Chrysostomou, Emma Lardner, Shu-Che Yu, Gustav Stålhammar, Jonathan G. Crowston, James R. Tribble, Peter Swoboda, Pete A. Williams

**Affiliations:** a Department of Clinical Neuroscience, Division of Eye and Vision, St. Erik Eye Hospital, Karolinska Institutet, Stockholm, Sweden; b Centre for Vision Research Duke-NUS & Singapore National Eye Centre, Singapore; c Save Sight Institute at the University of Sydney and Royal Prince Alfred Hospital, Sydney, Australia; d Department of Medicine Huddinge (MedH), Biosciences and Nutrition Unit, NEO, Karolinska Institutet, Huddinge, Sweden

**Keywords:** Sarcopenia, Metabolomics, Aging, Mitochondria, *C. elegans*

## Abstract

Sarcopenia is the age-related degeneration of skeletal muscle, resulting in loss of skeletal muscle tone, mass, and quality. Skeletal muscle is a source of systemic metabolites and macromolecules important for neuronal health, function, and healthy neuronal aging. Age-related loss of skeletal muscle might result in decreased metabolite and macromolecule availability, resulting in reduced neuronal function or increased susceptibility to unhealthy aging and neurodegenerative diseases. We aimed to identify muscle metabolite candidates that regulate healthy aging. C57BL/6J mice were aged to young adult (4 months) and old age (25 months) and skeletal muscle was collected. Age-related muscle loss was confirmed by reduced muscle mass, muscle fiber degeneration, reduced myosin intensity, in addition to a metabolic shift and increased DNA damage in skeletal muscle. Using a low molecular weight enriched metabolomics protocol, we assessed the metabolic profile of skeletal muscle from young adult and old age mice and identified 20 metabolites that were significantly changed in aged muscle. These metabolite candidates were tested in *C. elegans* assays of lifespan, healthspan, muscle, and mitochondrial morphology under normal and stressed conditions. We identified four metabolite candidates (beta-alanine, 4-guanidinobutanoic acid, 4-hydroxyproline, pantothenic acid) that, when supplemented in *C. elegans* provided robust gero- and mitochondrial protection. These candidates also affected life-, and health- span in *C. elegans* models of amyotrophic lateral sclerosis (ALS) and Duchenne muscular dystrophy (DMD). Our findings support that aging muscle can be used to identify novel metabolite modulators of lifespan and health and may show promise for future treatments of neurodegenerative and neuromuscular disorders.

## Highlights

• Metabolomics identified metabolites and macromolecules in young adult and aged mouse skeletal muscle tissue.
• Supplementation of β-alanine, 4-guanidinobutanoic acid, 4-hydroxyproline, and pantothenic acid extended lifespan in *C. elegans.*
• Supplementation of β-alanine, 4-guanidinobutanoic acid, 4-hydroxyproline, and pantothenic acid affected lifespan during oxidative and disease induced stress in *C. elegans.*
• Aging can molecularly be dissected with metabolite candidates. This work can serve as a tool for future metabolome screens.

## 1 Introduction

Sarcopenia is the age-related degeneration of skeletal muscle characterized by the progressive degeneration of muscle weight, tone and/or mass, resulting in reduced muscle capacity and strength. The prevalence of sarcopenia is 5–13 % for humans aged 60–70 and 11–50 % for those aged >80 years [1,2]. In sarcopenia, myofiber atrophy and a loss of myofiber numbers occurs, in addition to a loss of myofiber function due to neuromuscular junction destabilization [3]. Inflammation is a key hallmark of aging, as well as sarcopenia, and inhibition and deletion of a pro-inflammatory cytokine (IL-11) improved metabolic function and muscle quality in mice [4]. In aged muscle and sarcopenia, there is a greater loss of type II (fast twitch) than type I (slow twitch) muscle fibers, leading to an increased ratio of type I fibers and a respiratory switch, which exerts increased relaxation time and a loss of power [5,6]. This loss of power contributes to accidents, disability, and a poor quality of life in addition to correlating with cardiac and respiratory disease, cognitive impairment, and neurodegenerative disease [7].

Skeletal muscle is an important source and store of amino acids and regulator of metabolism by responding dynamically to bioenergetic demand [1]. To achieve these dynamic roles, muscle secretes amino acids, proteins, lipids, metabolites, cytokines (myokines), and small RNAs; collectively referred to as the muscle secretome [8]. Remaining physically active and high intensity training is not only an intervention for sarcopenia but also protects against neurodegenerative diseases. Supporting this, forced and voluntary exercise is profoundly neuroprotective in many animal models of neurodegenerative disease [[9], [10], [11], [12], [13]]. Together, this data supports the importance of systemic and active secretion of macromolecules and metabolites by skeletal muscle. Supplementation of macromolecules and metabolites and their effect on disease, healthspan and lifespan have therefore been an ongoing research topic and intervention strategy [[14], [15], [16]]. Since frailty correlates with the incidence of neurodegenerative diseases, identifying the molecules that are produced by the muscle that may drive aging and susceptibility to disease is of therapeutic importance [17].

In this study, we aimed to understand the relationship between skeletal muscle metabolomes and aging. The muscle metabolome of aged mice was used to identify altered metabolites with age and sarcopenia. Supplementation of these metabolite candidates in *C. elegans* extended lifespan, improved healthspan, and affected mitochondrial and myosin degeneration. The identified metabolite candidates also provided therapeutic benefit in *C. elegans* models of neuromuscular disease (amyotrophic lateral sclerosis; ALS, and Duchenne muscular dystrophy; DMD). Taken together, our study provides new strategies for gero- and metabolic-protection by targeting key metabolic pathways.

## 2 Results and discussion

### 2.1 Age-related or sarcopenic changes in mouse aged skeletal muscle

**Fig. 1:**
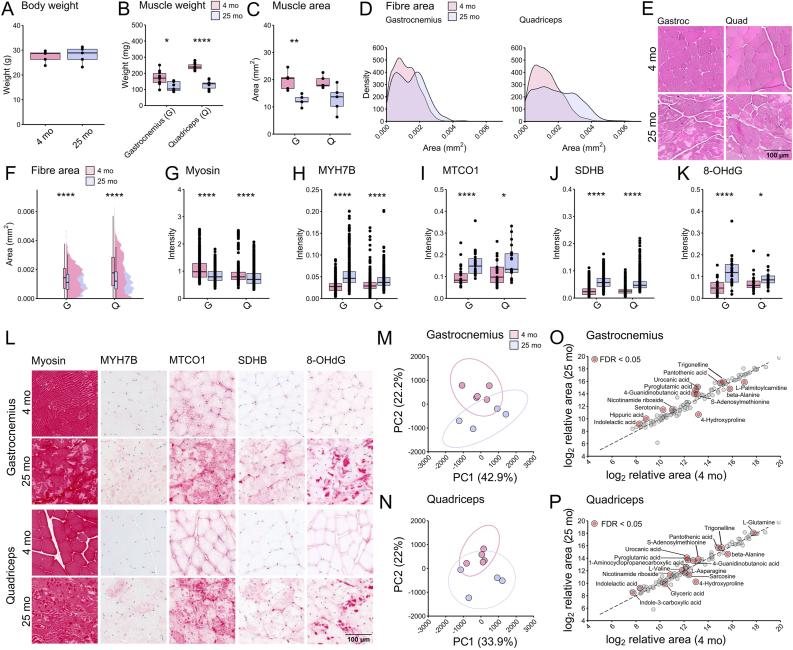
**Metabolic changes in aging murine skeletal muscle.** (**A-P**) C57BL/6J mice. (**A**) Body weight did not change during aging (n = 5/group). (**B**) Muscle weight and (**C**) area declined during aging, as did (**D-F**) fiber area, confirming sarcopenia. By histological assessment (**F-L**), it was observed that (**G**) myosin intensity declined while (**H**) MHY7B intensity increased, suggesting an increase in the ratio of type I (slow twitch) fibers. In line with this finding, (**I**) MTCO1, a component of cytochrome *c* oxidase, increased, together with (**J**) SDHB, a subunit of succinate dehydrogenase, suggesting a respiratory switch from glycolytic to oxidative. (**K**) 8-OHdG intensity increased, identifying cell death in aged tissue. (**M-N**) Unsupervised principal component analysis (PCA) separated samples into discrete groups that confirmed distinct metabolic profiles of young adult and aged muscle tissue. Statistical analysis of the low molecular weight metabolome identified 13 metabolites in (**O**) gastrocnemius and 17 in (**P**) quadriceps (significant hits in red). 10 were identical between the datasets, leaving 20 unique metabolites (see Table 1) (*t*-test FDR <0.05, volcano plot FDR <0.1). G: gastrocnemius, Q: quadriceps. *Pink* = 4 months of age, *purple* = 25 months of age. *p = ∗ < 0.05, ∗∗ < 0.01, ∗∗∗ < 0.001, ∗∗∗∗ < 0.0001.*

The age-related, or sarcopenic, loss of muscle mass is well-reported in human patients and model organisms [18]. However, there are clinically defined standards to diagnose sarcopenia in humans, but not in mice models, and it is therefore standard to analyse body and muscle weight in mice to define age related muscle loss, which may reflect sarcopenia in mice [19]. In a recent natural history study of sarcopenia, the body mass of male C57BL/6JRj mice was identified to be stable between 8 and 18 months, before it started to decline, and mice had lost ∼10 % of their body mass at 24 months of age [1]. To assess the metabolic profile of aged muscle we aged C57BL/6J (B6J) mice to 25 months (representing ∼70+ years of age in humans) [20]. At 25 months, B6J mice had unchanged body weight (Fig. 1A), whilst the weight and overall area of the skeletal muscles gastrocnemius and quadriceps decreased (Fig. 1B and C), with a concomitant muscle fibre degeneration (Fig. 1D–F). While the muscle degenerates, fat cells (adipocytes) infiltrate the muscle tissue, which associates to muscle dysfunction in disease- and aging states [21,22]. We identified adipocyte infiltration in skeletal muscle, which was more prevalent in the quadriceps than in the gastrocnemius (Fig. S1A and B). The gastrocnemius and quadriceps are skeletal muscles, located in the lower leg and front of the thigh, and are characterized by a high proportion of type II, fast-twitch fibres that have high glycolytic capacity to rapidly generate ATP [23]. During aging and in sarcopenia, both type I and type II fibres degenerate but with a preference for type II fibre degeneration [24]. Fibre types are generally characterized by the expression of myosin heavy chain (MyHC) isotypes [25], and we confirmed sarcopenia, by a decrease of myosin, representing both type I and II fibres, whilst MYH7B increased (type I fibre specific myosin (Fig. 1G and H), suggesting an increase in the proportion of type I fibres.

Mitochondrial density and function are of great importance in skeletal muscle, generating ATP rapidly at sites of action and accumulating at neuromuscular junctions (NMJs) [26,27]. Mitochondrial degeneration is a hallmark of sarcopenia and a target for treatment strategies in sarcopenia [28]. We next assessed gross metabolic potential using histopathological techniques (Fig. 1L). Cytochrome *c* oxidase is a biomarker for mitochondrial injury, oxidative stress and ROS production and apoptosis and is often used to diagnose mitochondrial disease [29,30]. Histopathology of skeletal muscle demonstrated an increase in MTCO1 intensity (cytochrome *c* oxidase; mitochondrial Complex IV) (Fig. 1I) but no change in CYOC intensity (Cytochrome C Oxidase subunit VIc/COX6C) of cytochrome *c* oxidase (Fig. S1A and D) in aged skeletal muscle tissue, demonstrating mitochondrial injury. Succinate dehydrogenase complex iron sulfur subunit B (SDHB), a protein subunit of succinate dehydrogenase (mitochondrial Complex II), increased during aging (Fig. 1J), while this generally decreases during aging [31]. The observed respiratory switch in sarcopenic muscle by the loss of glycolytic (type II) fibres and an increased ratio of oxidative (type I) fibres could explain the observed increase in complex II and increased oxidative respiration. No differences were observed for Tomm20, a complex that is involved in protein transport into mitochondria and used as a marker for mitochondrial numbers or accumulation, demonstrating no gross difference in mitochondrial mass/numbers (Fig. S1A and C).

DNA damage is a common aging hallmark [32]. DNA damage was characterized by the presence of 8-OHdG, targeting hydroxy deoxyguanosine, a modified base in DNA due to attack by hydroxyl radicals (formed as byproducts of aerobic metabolism and ROS), demonstrating an increase of DNA damage in aged muscle tissue (Fig. 1K). Collectively, this data demonstrates aged tissue and metabolic compromise in the skeletal muscle of 25-month-old mice.

### 2.2 Low molecular weight metabolomics of mouse skeletal muscle identifies altered metabolism

We next conducted metabolomic profiling of young adult and aged muscle. We utilized a low molecular weight metabolomics platform to measure 502 low molecular weight metabolites in skeletal muscle [33]. The resulting skeletal muscle metabolomes demonstrated a clear change with advancing age. Principal component analysis confirmed distinct metabolic profiles of young adult and aged muscle tissue but showing variation between samples by PC1 (principal component 1) and PC2 (Fig. 1M and N). In the gastrocnemius tissue, 13 metabolites significantly changed with advancing age, and 17 metabolites significantly changed in the quadriceps tissue. Between the gastrocnemius and quadriceps datasets, 10 metabolites were significantly changed in both muscle tissues, and 20 were unique to one muscle group (Fig. 1O and P, Table 1). KEGG (Kyoto Encyclopedia of Genes and Genomes) enrichment analysis identified *arginine and proline*-; *beta-alanine-;* *histidine-;* *glycine,* *serine and threonine-*; *glyoxylate and dicarboxylate-;* *nicotinate* *and* *nicotinamide metabolism;* and *pantothenate and CoA biosynthesis* as the most affected pathways. *Pantothenate and CoA biosynthesis* were identified as significant, leaving the other pathways as matched with the identified metabolites but not predicted to be significantly changed (Fig. S1E). RaMP DB (Relation database of Metabolic Pathways) enrichment identified several transport processes as neurotransmitter transporters, transport of small molecules, transmembrane transport, and metabolic processes as histidine catabolism and metabolism, metabolism of amino acids and derivatives, and metabolic disorders of biological oxidation enzymes (Fig. S1F).

**Table 1:** Significant hits and assessment of metabolites.

Metabolite	FC Gastroc	FC Quad	Effect	Previously identified/Effect	Novel
L-Aminocyclopropanecarboxylic acid		1.2	↑		Yes
L-Asparagine		1.3	↑	Yes/lifespan increase	
β-alanine	0.5	0.5	↓		Yes
L-Glutamine		1.3	↑	Yes/lifespan increase	
Glyceric acid		0.7	↓		Yes
4-Guanidinobutanoic acid	2.3	1.8	↑		Yes
Hippuric acid	2.3		↑		Yes
4-Hydroxyproline	0.2	0.1	↓		Yes
4-Hydroxyphenylpyruvic acid					Yes
Indoleacetic acid (auxin)	1.9	1.9	↑	Yes/lifespan increase	
Indole-3-carboxylic-acid		1.6	↑		Yes
Nicotinamide riboside	1.4	1.4	↑	Yes/lifespan increase	
L-Palmitoyl carnitine	0.5		↓		Yes
Pantothenic acid	1.7	1.8	↑		Yes
Pyroglutamic acid	3.3	2.7	↑		Yes
S-Adenosyl methionine	1.8	1.5	↑	Yes/lifespan increase	
Sarcosine		0.5	↓		Yes
Serotonin	2.3		↑	Yes/lifespan increase	
Trigonelline	1.6	1.5	↑	Yes/lifespan increase	
Urocanic acid	4.0	3.6	↑		Yes
L-Valine		0.7	↓	Yes/lifespan increase	

Metabolites identified from murine muscle in the low molecular weight metabolomics dataset were assessed for follow-up studies in *C. elegans* assays. Literature- and pathway analysis was performed for each significant hit (p-value and fold-change (FC), 4-Hydroxyphenylpyruvic acid was not identified by volcano plot but was included based on FC). Literature analysis was performed in PubMed and Textpresso (search strategy by metabolite + mice/*C. elegans*/lifespan), and pathway analysis in *C. elegans (*KEGG and wormflux) for each metabolite [50,51] Literature analysis demonstrated that eight metabolites had extensive literature related to longevity in mice and/or *C. elegans*; lifespan effects of metabolite candidates were well studied and published. The previously identified and novel candidates were further triaged based on availability and cost; all candidates were commercially available. All candidates were followed up for lifespan extending effects in *C. elegans.*

### 2.3 Triaging of low molecular weight metabolites identified enhancers of life and healthspan in C. elegans

We next assessed the 20 metabolite candidates, based on existing literature and pathway analysis, in lifespan assays in *C. elegans,* a short-lived model organism that is widely used for lifespan analysis [34,35]. l-Asparagine, l-glutamine, auxin, nicotinamide riboside, S-adenosyl methionine, serotonin, trigonelline, and l-valine had been examined before in *C. elegans* assays (Table 1). Despite previous examination, all 20 candidates were investigated in *C. elegans*; where a flooding assay was performed (*C. elegans* were flooded with M9 buffer, worms that thrash/float identify as live/dead) at day 16 of adulthood to initially screen for potential lifespan enhancers (Table S4). All supplementation was performed from day 1 of adulthood (young adults) to not interfere with larval development and to model supplementation in humans which is usually not done before reaching adulthood. Wild-type N2 *C. elegans* worms are considered young adults at day 2 of adulthood, start degenerating around day 8, reach old age around day 12 and very old age beyond day 16 (this is matched in our laboratory) [36,37].

Glyceric acid (GA), 4-guanidinobutanoic acid (4GBA), 4-hydroxyproline (4HP), Indole-3-carboxylic-acid (I3CA), nicotinamide riboside (NR) and L-palmitoyl carnitine (LPC) demonstrated increased live/dead ratios at day 16 compared to vehicle (sterile double distilled (dd)H2O (2.5 %) or DMSO (2.5 %)), while only LPC was statistically significant (Fig. S2A). However, LPC treated worms were significantly smaller and showed decreased locomotion from day 2 of adulthood indicating decreased overall health (Fig. S2B); and were thus excluded from all further experimentation. Based on this data, long-term survival assays were performed for the top four of novel metabolite candidates with lifespan and neurodegenerative and neuromuscular enhancing potential: β-alanine, 4-guanidinobutanoic acid, 4-hydroxyproline and pantothenic acid. These four are of specific interest regarding neurodegenerative and neuromuscular disease since beta-alanine is a component of carnosine*,* 4-guanidinobutanoic acid is synthesized to 4-amino butanoate (GABA), 4-hydroxyproline is synthesized into pyruvate, and pantothenic acid into coenzyme A.

**Fig. 2:**
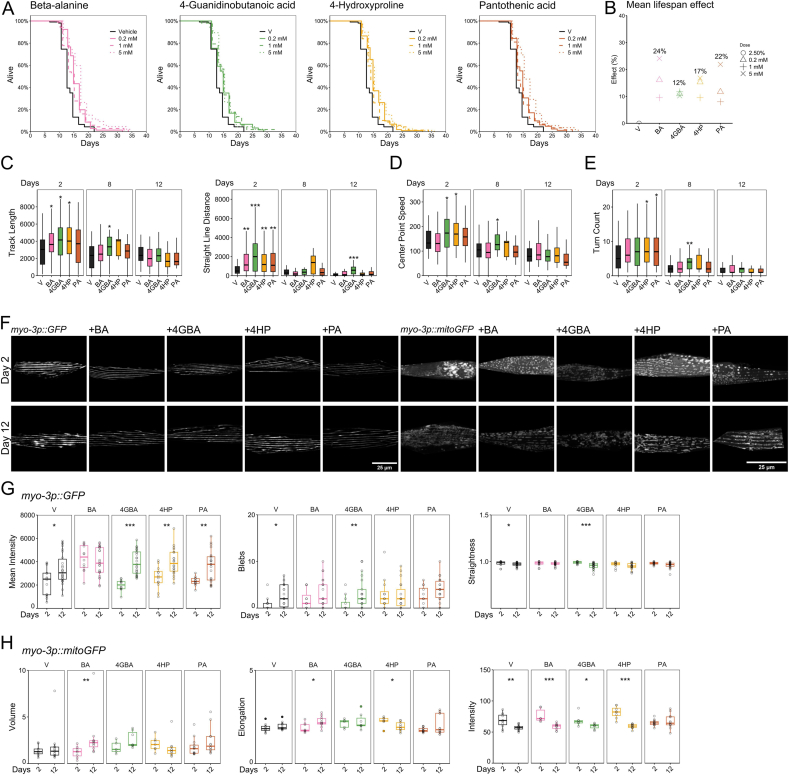
**Identification of novel metabolite candidates for gero- and metabolic protection in *C. elegans***. (**A-D**) *C. elegans*, wild-type N2, (**E-F**) *C. elegans* strains RW1596 and SJ4103 (cf. Suppl. Table S2). (**A**) Based on literature and pathway analysis, we identified 4 top hits, which all increased lifespan at 0.2, 1 and 5 mM, lifespan is shown in days. (**B**) Based on the survival curve, the mean lifespan effect was identified, which ranged from 10 to 25 %. (**C**) Healthspan analysis after 5 mM supplementation from day 1 of adulthood by locomotion (track length (center point trajectory), peristaltic track length (forward – reverse), peristaltic speed (forward – reverse) and straight line distance) (WormLab, MBF Bioscience) decreased between day 8 and day 12 of adulthood, 4GBA supplementation movement increased movement at day 12. (**D**) Fluorescence microscopy images representing *myo-3p::GFP* at day 2 and day 12 of adulthood, and confocal microscopy images representing *myo-3p::mitoGFP* at day 2 and day 12 of adulthood. During aging, myosin fibres degenerate, lose straightness and increase in the number of blebs. Mitochondrial degeneration is observed by a loss of mean intensity. (**E**) Myosin fibres after 5 mM supplementation from day 1 of adulthood were quantified by MuscleMetrics, plots represent the mean intensity, number of blebs and straightness for all supplements at day 2 and day 12 (*n = number of sarcomeres:* VH = 14/26, BA = 12/23, 4GBA = 12/25, 4HP = 12/20, PA = 12/22). (**F**) Mitochondria after 5 mM supplementation from day 1 of adulthood were quantified by Imaris and MitoAnalyzer, plots represent the volume, elongation, and intensity for all supplements at day 2 and day 12 (*n = number of worms, average of 2 regions* per *wormat day 2/day 12:* VH = 11/14, BA = 6/10, 4GBA = 6/7, 4HP = 8/11, PA = 14/12). V: vehicle, BA: β-alanine, 4GBA: 4-guanidinobutanoic acid, 4HP: 4-hydroxyproline, PA: pantothenic acid*. p = ∗ < 0.05, ∗∗ < 0.01, ∗∗∗ < 0.001, ∗∗∗∗ < 0.0001.*

Supporting our triaging strategy, the novel candidates β-alanine (BA), 4-hydroxyproline (4HP), 4-guanidinobutanoic acid (4GBA) and pantothenic acid (PA) extended *C. elegans* lifespan at 0.2, 1 and 5 mM supplementation from day 1 of adulthood (Fig. 2A and B, Table S5). *C. elegans* supplementation studies generally require much higher doses than *in vitro* cell culture studies due to the *C. elegans* cuticle (where the only means of supplemented molecules entering the body are the pharynx, vulva and anus) and it remains unknown how much of a supplemented molecule reaches the inside body. Used doses were based on a previous amino acid supplementation study [38]. While BA and 4HP demonstrated decreased levels in the mouse aged skeletal muscle tissue, 4GBA and PA demonstrated increased levels, and the observed ratios were like previously published results [2]. Despite the opposite skeletal muscle levels, supplementation of 0.2, 1 and 5 mM of the metabolite candidates increased lifespan in *C. elegans*, however in a non-dose-dependent manner where 0.2 and 5 mM had the most extensive effects on lifespan. Lifespan increases are not always dose-dependent in *C. elegans,* which was reflected by the amino acid supplementation study where it was hypothesized that the rate of transport influences the effects [38]. Although *C. elegans* have high genetic homology to humans, not all metabolic pathways are identical or use the same metabolic intermediates. In the metabolomics screen we removed metabolites that are known or predicted to be of no relevance in *C. elegans*. As such, it is perhaps not unexpected that some metabolites have no, or opposite predictive, effects when translated from mammalian to nematode systems.

We next assessed several parameters during aging and with supplementation of BA, 4GBA, 4HP, and PA which included healthspan by locomotion, myosin morphology (to study a sarcopenic state), mitochondrial morphology (since many neurodegenerations have a typical metabolic hallmark), and oxidative stress resistance (aging and neurodegenerations demonstrate increased oxidative stress) and in a selection of disease models (ALS and DMD) based on our hypothesis of an active exchange of molecules between skeletal muscle and the CNS. *C. elegans* healthspan measurements often include preservation of locomotion, feeding behaviour and accumulation of auto-fluorescent pigment. For example, it has previously been reported that short-lived *C. elegans* mutants score worse on locomotion parameters like speed and amplitude [39]. Of note, a consensus on parameters to determine healthspan in *C. elegans* is lacking. However, it was recently suggested that healthspan can broadly be determined by measuring physiological parameters like oxidative stress resistance, heat stress resistance, thrashing, and distance travelled [40].

We first established a clear aging phenotype in wild type worms, as recorded and analysed by WormLab (MBF Bioscience), which identified by hierarchical clustering (Morpheus, Broad Institute) that several parameters demonstrate a clear decrease during aging; including reduced track length (center point trajectory), peristaltic track length (forward movement – reverse movement), peristaltic speed (forward – reverse) and straight-line distance between day 2 and 20 of adulthood (these days were used to note obvious differences between young adult and old aged worms, further experiments include middle aged to aged worms; day 5 to day 12) (Fig. S2D). This aging phenotype was then verified in *C. elegans* models of neuromuscular disease (amyotrophic lateral sclerosis; ALS and Duchenne muscular dystrophy; DMD) (Fig. S3A).

We supplemented the metabolite candidates to *C. elegans* during a 20-day period with 5 mM of metabolite candidate (5 mM had the most extensive effects on survival) (starting at day 1 of adulthood), and although small increases of activity were noted, 4GBA caused consistently significant effects (at day 12 of adulthood) on all the three identified locomotion parameters (peristaltic track length, speed (forward – reverse), and straight-line distance), while 4HP had minor locomotion increasing effects at day 8 of adulthood (Fig. 2C). To interfere with endogenous levels of metabolite candidates, RNA interference-by-feeding was performed against genes in the 4-hydroxyproline pathway. Straight-line distance decreased between day 2 and 8 of adulthood, while supplementation of 4-hydroxyproline partly recovered this locomotion (Fig. S2E). Interference against *dpy-18* and *phy-2*, which synthesize l-proline to 4-hydroxyproline, decreased this recovery effect as did interference against *pycr-2* (4-hydroxyproline to L-1-pyrroline-3-hydroxy-5-carboxylate) to an even greater extent (Fig. S2E). 4-Hydroxyproline is further metabolized into pyruvate and glyoxylate; potentially explaining the observed lifespan and locomotion enhancing effects of 4-hydroxyproline, while RNAi in this pathway decreased locomotion. Pyruvate has previously been identified to play an important role in the lifespan extension in *C. elegans* after dietary restriction (DR) where pyruvate levels were increased [41] and pyruvate supplementation has demonstrated to increase *C. elegans* lifespan and its oxidative stress resistance [42].

### 2.4 Gero protection demonstrated by mitochondrial and myosin protection

Mitochondrial degeneration within skeletal muscle is one of the first hallmarks of sarcopenia. We studied the effect of BA, 4HP, 4GBA, and PA on skeletal muscle and their mitochondria using *C. elegans* that express GFP (green fluorescent protein) under the *myo-3p* (myosin heavy chain) gene promotor, or that express GFP only in mitochondria, under the same promotor (Fig. 2D). Recent studies have predicted biological age based on sarcopenia in *C. elegans* where ‘delayed onset of sarcopenia served as a biomarker of extended lifespan’ [43]. Using this toolbox, we identified that BA preserved several parameters (GFP intensity, number of blebs, muscle straightness), supporting a delayed onset of sarcopenia. 4GBA showed a similar effect on all parameters as the vehicle condition, suggesting an early sarcopenic state at day 12 of adulthood, whilst 4HP and PA scored similar as vehicle on some but not on all parameters (Fig. 2D, E, Fig. S2F). BA is synthesized into carnosine, and BA supplementation has demonstrated to increase carnosine content in muscle [44]. Carnosine content is high in the brain and muscle and has demonstrated positive effects on muscle by affecting ion chelation, ROS scavenging, and calcium handling [45].

Mitochondrial networks are tightly regulated and change during aging. Mitochondrial networks can consist of small, fragmented units or of larger elongated networks, and it has previously been reported that mitochondria elongate during nutrient deprivation, protecting them from autophagy [46]. Mitochondrial volume and network elongation increased following BA supplementation, demonstrating a larger, more elongated network. PA preserved mean GFP intensity. 4HP decreased mitochondrial elongation, while increasing the number of surfaces and sphericity, suggesting a network of smaller and round units (Fig. 2D–F, Fig. S2G). Preserved GFP intensity by PA demonstrates preservation of mitochondria (but importantly, presence of GFP alone does not comment on the function of the mitochondrion itself). PA is synthesized to coenzyme A (CoA) with cysteine and ATP via four enzymatic steps (in humans and *C. elegans*). CoA is the building block to many metabolic processes as fatty acid and lipid metabolism, and ferroptosis; mechanisms that are affected in both neurodegenerative disease and aging [47,48]. 4HP is synthesized into pyruvate and glyoxylate and the observed network of smaller and round units hints to altered mitochondrial dynamics; pyruvate supplementation previously demonstrated altered mitochondrial dynamics [49]. Future experiments are needed to determine the functional state of aged mitochondria after metabolite candidate supplementation.

### 2.5 Gero protection demonstrated by induced disease and oxidative stress

**Fig. 3:**
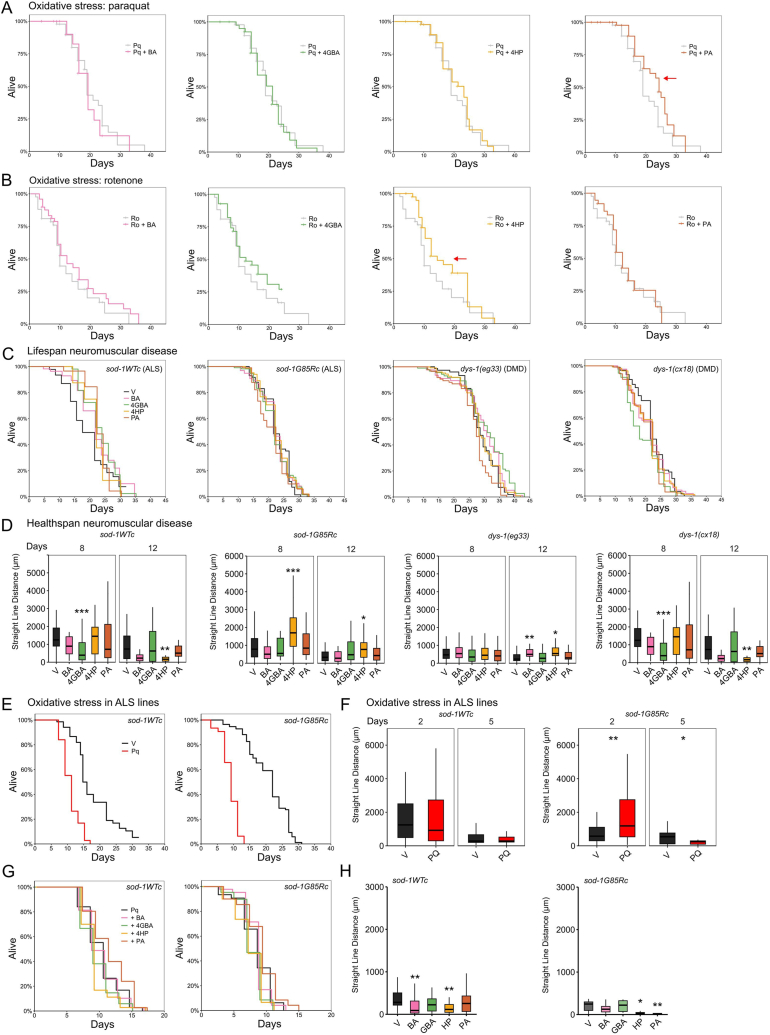
Metabolite candidates overcome oxidative and disease stress in *C. elegans.* (**A, B**) *C. elegans*, wild-type N2, (**C, D**) *C. elegans*, ALS models (strains HA2986 (*rt448*) and HA3299 (*rt451*)) and DMD models (strains BZ33 (*eg33*) and LS292 (*cx18*)). All metabolite candidates were supplemented at 5 mM. (**A**) Low dose oxidative stress induced by paraquat (16 mM, single dose at day 1 of adulthood) extended lifespan, which was rescued by 5 mM of PA, but not by the other metabolite candidates. **(B**) Oxidative stress induced by rotenone (2 μM, single dose at day 1 of adulthood) was overcome the metabolite candidate 4HP (p-values are listed in Table S6). (**C**) Metabolite supplementation did not decrease or increase lifespan in an ALS *sod-1* WT^C^ and -G85R^C^ strain. 4GBA supplementation increased lifespan in the DMD model (*eg33*), but decreased lifespan in (*cx18*), as did PA supplementation, (p-values are listed in Table S7). (**D**) Healthspan analysis by locomotion (straight line distance) identified 4HP as a locomotion modulator in the ALS *sod-1* G85R^C^ model, but not in the WT^C^ model and BA as a locomotion modulator in the DMD model (*eg33*). (**E**) Lifespan analysis demonstrated reduced lifespan of paraquat (16 mM, re-supplemented every 2 days) treated WT^C^ and G85R^C^ compared to vehicle condition. (**F**) Locomotion analysis at day 5 of adulthood by straight line distance demonstrated decreases for paraquat treated *sod-1* G85R^C^. (**G**) Lifespan analysis demonstrated decreased lifespan for paraquat + 4HP and 4GBA treated *sod-1* WT^C^ and G85R^C^, none of the metabolite candidates saved paraquat induced decreased lifespan. (**H**) Locomotion analysis at day 5 of adulthood by straight line distance demonstrated decreased locomotion for paraquat + BA and 4HP treated WT^C^ and G85R^C^, none of the metabolite candidates saved paraquat induced decreased locomotion. V: vehicle, BA: β-alanine, 4GBA: 4-guanidinobutanoic acid, 4HP: 4-hydroxyproline, PA: pantothenic acid*. p = ∗ < 0.05, ∗∗ < 0.01, ∗∗∗ < 0.001, ∗∗∗∗ < 0.0001.*

As a broad healthspan analysis involves oxidative stress resistance, we supplemented the metabolite candidates in models of oxidative stress. Wild-type N2 worms were exposed to rotenone and paraquat, which are both inducers of metabolic/oxidative stress by inhibition of mitochondrial Complex I, redox cycling, and by the production of reaction oxygen species (ROS) [50,51]. Under metabolite supplementation, PA increased survival in the paraquat model of ROS stress, whilst 4HP increased lifespan in the rotenone model (inhibition of Complex I), supporting that metabolite candidates affect different mitochondrial processes. This observation is supported by the mitochondrial morphological data, where we observed altered mitochondrial dynamics by PA and 4HP in alternating ways, potentially exerted via the downstream metabolites pyruvate and CoA (Fig. 3A and B, Table S6). Future experiments could explore the effects of pyruvate and CoA supplementation.

Since the metabolite candidates were able to partially overcome induced metabolic stress, we also assessed their effects in connection to more chronic neuromuscular disease. We therefore applied the candidates in *C. elegans* models of ALS and DMD (Table S2). To assess these candidates in the context of ALS we used *sod-1*G85R^C^ mutant and *sod-*1WT^C^ wild-type strains, where the *sod-1*G85R^C^ mutant is a single copy ALS SOD1 knock-in model, which exhibits both cholinergic and glutamatergic neuron degeneration with an increased sensitivity to paraquat exposure [52]. Survival assays identified that BA, 4GBA, 4HP, and PA slightly increased mean lifespan of the *sod-1*WT^C^ wildtype strain, but not in the *sod-1*G85R^C^ mutant, with PA decreasing lifespan in the knock-in mutant strain (Fig. 3C–S3A-B, Table S7)**.** 4HP, caused consistent significant increase of healthspan by locomotion at day 8 and 12 of adulthood in the G85R^C^ strain, but not in the WT^C^ strain (Fig. 3C–S3B). The *sod-1*G85R^C^ mutant is paraquat sensitive due to a mutation in superoxide dismutase (SOD) which results in lower enzymatic activity in destroying or neutralizing oxidative species. 4HP did not demonstrate any effects in the *sod-*1WT^C^ worms or on paraquat supplementation in wildtype N2 worms but showed increased motility in the s*od-1*G85R^C^ worms potentially via pyruvate; pyruvate has been demonstrated to increase motor performance in G93A SOD1 mice [53]. To assess DMD, *dys-1*(*eg33*) and *dys-1*(*cx18*) mutant DMD strains were used, with *dys-1*(*eg33)* demonstrating impaired mitochondrial network integrity and function and a more clinically relevant phenotype than *dys-1*(*cx18)* [54]. BA and 4GBA slightly increased mean lifespan in the *dys-1*(*eg33*) background, whereas PA decreased lifespan. BA and 4HP had the most effect on healthspan by increased locomotion in the *eg33* strain but not in the *cx18* strain (Fig. 3D–S3B, Table S7). BA has been suggested as a nutraceutical for DMD as it exerts ROS scavenging effects. BA supplementation in a DMD mouse model increased “resistance to fatigue after intermittent electrical stimulation” [55]. The *dys-1*(*eg33*) demonstrates impaired mitochondrial network integrity and 4HP altered mitochondrial dynamics (increased motility). Future experiments could utilize -omics studies before/after supplementation in the disease models to mechanistically understand and begin to confirm how the metabolite candidates exert their effects.

Since many neurodegenerative conditions are multifactorial, where both genes and stressors play a role in disease initiation and pathology, we stressed the ALS *sod-1*WT^C^ and *sod-1*G85R^C^ mutant strains with paraquat and applied simultaneous supplementation to identify protection by the metabolite candidates. In line with previous studies, healthspan and lifespan decreased after paraquat exposure, which was more prominent in the G85R^C^ mutants (Fig. 3E–F, Fig. S3C, Table S8) [52]. Metabolite supplementation did not induce survival, instead BA, 4GBA and 4HP decreased survival and healthspan in WT^C^ and G85R^C^ models exposed to paraquat stress, while PA yielded similar results as paraquat (Fig. 3G, H, Fig. S3D, Table S8). This is in line with our observation that PA was the only metabolite that could rescue paraquat induced stress in wild-type N2. Supplementation at 5 mM was not able to counteract the multifactorial stress. Future studies could explore repeated dosing, an increased dose of metabolite candidates, or combined treatment of metabolite candidates against multifactorial stress.

## 3 Conclusion

In conclusion, we observed a significant decline of mouse skeletal muscle during aging, which was reflected by an altered skeletal muscle metabolome at an old age. Significantly altered low molecular weight metabolites can impact aspects of longevity and mitochondrial health when supplemented in *C. elegans.* While all the final metabolite candidates analysed (BA, 4GBA, 4HP, PA) affected lifespan, none of them impacted all the parameters studied: lifespan, healthspan, mitochondrial phenotype, and myosin phenotype, oxidative stress, and disease stress. BA demonstrated an effect on myosin morphology and motility in the DMD model with an impaired mitochondrial network; 4HP altered mitochondrial networks and resistance against oxidative stress in an induced stress model of rotenone exposure and mutant SOD; 4GBA demonstrated lifespan and locomotion enhancing effects; and PA demonstrated effects on mitochondrial preservation and resistance against induced oxidative stress by paraquat. This data suggests that a metabolite candidate can show significant effects in one aspect but not in the other. This demonstrates that our successful muscle metabolome screen isolated specific compounds for (only) specific phenotypic and biological aspects of aging. Thus, our work supports that aging (life and healthspan) can molecularly be dissected through metabolite candidates, as these target specific aspects and leave others untouched. Consequently, this approach can serve as a template and tool for future metabolome screens in healthy or diseased conditions.

## 4 Methods

### 4.1 Mouse strains, breeding and husbandry

All animal procedures conformed to the National Research Council's Guide for the Care and Use of Laboratory Animals and were approved by the SingHealth Institutional Animal Care and Use Committee (2019/SHS/1534). C57BL/6J mice were purchased from InVivos Pte Ltd, Singapore, and housed at the SingHealth Experimental Medicine Centre (Academia, Singapore) in a temperature- (22 ± 1 °C), light- (12 h light, 12 h dark) and humidity-controlled (30–40 %) environment with free access to food and water. Male and female C57BL/6J mice were aged to young adult (4 months, n = 5) and old age (25 months, n = 5) after which they were euthanized by cervical dislocation.

### 4.2 Histology: skeletal muscle

After euthanizing mice, the gastrocnemius and quadriceps were immediately dissected in ice cold HBSS, wiped dry, weighed, and frozen on dry ice. Tissue was stored at −80 °C and shipped, kept in dry ice, to Karolinska Institutet for histological sample processing and to the Swedish Metabolomics Centre for metabolomic sample processing. 3 μM muscle sections were prepared with a vibratome, followed by embedding in paraffin wax. Sections were placed on glass slides and baked at 60 °C for 1 h. Immunohistochemistry (IHC) was performed as previously described [56]. Briefly, an automated IHC machine (Leica) was used to perform chromogenic IHC. To avoid batch effects, samples labelled with the same antibody were processed as one batch (list of antibodies in Table S1). Wax sections were deparaffinized, dehydrated and antigen was retrieved. Sections were washed and incubated in primary antibody, followed by washing and incubation in a polymer conjugated secondary antibody and colour development. Sections were dehydrated, cleared, mounted, and covered with a glass coverslip.

### 4.3 Imaging and analysis: skeletal muscle

Whole muscle slices were scanned at 400 × with a Grundium Ocus 40 scanner (Grundium Oy, Tampere, Finland). H&E staining was used to analyse total muscle area and muscle fibre area, and to determine five regions of interest (ROIs, 2500 μM^2^). Within the ROIs, fibres were quantified by segmentation using Cell Pose [57]. For fat infiltration quantification, H&E images were manually analysed on adipocyte infiltration by selecting adipocytes cells and -clusters within the whole muscle area. In QuPath, ROIs were analysed on intensity measurements (with background subtraction) for myosin, MHY7B and SDHB. Intensity measurements of single muscle fibres inside the five ROIs were analysed for Tomm20, CyoC, MTCO1, and 8-OHdG [58].

### 4.4 Low molecular weight metabolomics: skeletal muscle

Low molecular weight metabolomics was performed as previously described [33]. Briefly, extraction buffer including internal standards were added to the muscle sections together with 1 tungsten bead. Tissues were shaken in a mixer mill and samples were then centrifuged. Supernatant was transferred to micro vials and evaporated to dryness in a speed-vac concentrator. Samples were stored at −80 °C and small aliquots of the remaining supernatants were pooled and used to create quality control (QC) samples. Prior to the analysis, samples were re-suspended in 10 + 10 μL methanol and elution solvent A. The samples were analysed in batches according to a randomized run order. Each batch of samples was first analysed in positive mode. After all samples within a batch had been analysed, the instrument was switched to negative mode and a second injection of each sample was performed. The chromatographic separation was performed on an Agilent 1290 Infinity UHPLC-system (Agilent Technologies, Waldbronn, Germany) and compounds were detected with an Agilent 6546 Q-TOF mass spectrometer equipped with a jet stream electrospray ion source operating in positive or negative ion mode. MSMS analysis was run on the QC samples for identification purposes. All data pre-processing was performed using the Agilent MassHunter Profinder version B.10.0 SP1 (Agilent Technologies Inc., Santa Clara, CA, USA). The quantification of the metabolites was calculated as area under the curve of the mass spectrometry peak and normalized first to an internal standard for negative and positive runs, then for the weight of the tissue. Data were analysed and graphs were made using MetaboAnalyst (version 5.0; 28, 29) and R. All data were subject to Pareto scaling. Hierarchical clustering (HC) (Spearman, Average) was used to create the dendrograms. Comparisons between groups were analysed by two-sample *t*-tests with an adjusted p-value (false discovery rate, FDR), using a cutoff of 0.05 considered significant. Quantitative pathway analysis was performed using the *Mus musculus* KEGG library in MetaboAnalyst 5.0. One animal was excluded from analysis due to outliers.

### 4.5 *C. elegan*s strains and maintenance

*C. elegans* strains (Table S2) and *E. coli* OP50 bacterial food were obtained from the Caenorhabditis Genetics Centre (CGC, https://cgc.umn.edu/). Strains were maintained on 6 cm dishes at 20 °C on nematode growth medium (NGM) with *E. coli* OP50, both prepared according to standard protocols. Chemicals used in *C. elegans* assays are listed in Table S3.

### 4.6 Longevity and healthspan: *C. elegans*

All assays were performed on 6 cm dishes with nematode growth medium agar (NGM) and at 20 °C. NGM was prepared according to standard protocols. To prevent the generation of offspring and inhibit egg hatching, 50 μM of FuDR was added to NGM. *E. coli* OP50 as a food source was prepared according to standard protocols, after which it was treated with 1 % PFA for 1 h to metabolically inactivate bacteria [59]. After 1 h, OP50 was washed four times with sterile PBS, followed by a 10x concentration in LB and 100 μL per plate was seeded onto NGM plates. Plates were dried at RT for 24 h in the dark.

For the flooding assays, metabolite candidates were prepared by dissolving in distilled H_2_O or DMSO and after which they were diluted to 5 mM in OP50 treated with 1 % PFA and 100 μL per plate was seeded. For the survival, healthspan, oxidative stress and disease stress assays, metabolite candidates were freshly prepared and dissolved in distilled H_2_O or DMSO and were sterile filtrated. Plates were prepared by supplementing NGM agar with 0.2 mM, 1 mM or 5 mM of a metabolite candidate. Vehicle conditions consisted of 2.5 % of distilled H2O or 2.5 % of DMSO. Plates were prepared using a peristaltic pump (9 mL) and were dried at RT for 24 h in the dark.

For all assays, a timed egg lay was performed where gravid adults laid eggs for 4–5 h on NGM plates with live OP50. After 4–5 h, gravid adults were removed. At day 1 of adulthood, L4 to young adult animals were transferred to treatment/supplemented plates (minimum of 10 and maximum of 30 worms per plate). For flooding assays, at day 16 of adulthood, 2 mL of M9 buffer was added to the plates and worms were scored as alive (A) when thrashing was visible and scored as dead (D) if worms did not make movements (after poking) and/or were stuck to the plate. Missing worms and worms showing signs of internal hatching were censored (C) (4 independent experiments with a minimum of 2 plates per condition were performed. For survival assays, worms were scored for live, dead, censored status every 2–4 days. Worms were scored as alive (A) when a reaction to gentle poking with a worm pick was visible and scored as dead (D) if worms did not respond to a worm pick. Missing worms and worms showing signs of internal hatching were censored (C). A log-rank test or *Cox* proportional hazards model was performed to calculate survival estimates. For healthspan assays, worms were tracked in real-time on seeded NGM agar plates by the WormLab system (MBF Biosciences). Plates were gently tapped to stimulate movement and 30 s videos (14 frames per second) were recorded of a selection of worms. For every independent experiment, a minimum of two NGM plates per condition was used. The number of independent experiments is listed in the supplementary tables. To assess an aging phenotype, hierarchical clustering by Euclidian distance (Morpheus, Broad Institute; https://software.broadinstitute.org/morpheus/) was performed on Wormlab recorded data from day 2 and day 20 of adulthood. Data was first log_2_ adjusted. One datapoint was excluded because it was an outlier.

### 4.7 RNA interference: *C. elegans*

*E. coli* HT115(DE3) clones containing dsDNA plasmid inserts for *dpy-18* (Y47D3B.10), *phy-2* (F35G2.4), and *pycr-1* (M153.1) were obtained from the Julie Ahringer feeding library (including positives for lethality (*pos-1*) and a movement disorder (*unc-112*)). Bacterial clones were grown for 12 h in LB + 25 μg/ml ampicillin after which the grown colonies were 4x concentrated before seeding 100 μL per plate. L3 wild-type (N2) worms were placed on 1 mM IPTG (+25 μg/ml carbenicillin) plates with the respective HT115(DE3) clones of interest. The L4 stage F2 generation was used for experimental plates were 5 mM 4HP or vehicle was supplemented in the NGM (+IPTG, carbenicillin and 50 μM FuDR). On day 8 of adulthood, videos of worms were recorded with the Wormlab system (MBF Bioscience).

### 4.8 Chemical and oxidative stress: *C. elegans*

NGM plates were prepared as described above, paraquat (16 mM) and rotenone (2 μM) were dissolved in 1 % PFA OP50 and seeded on 5 mM supplemented plates. For chemical stress assays with wild-type N2, worms were transferred to (fresh) stress plates at day 1 of adulthood and followed up until death. For chemical stress assays with ALS sod-1 worms (*sod-1*WT^c^ and *sod-1*G85R^c^), worms were transferred to fresh plates at day 1, 3 and 5 of adulthood (metabolite candidates were dissolved in NGM and paraquat in OP50 treated with 1 % PFA) and paraquat in 100 μL dead OP50 was added every two days from day 7 of adulthood till death. Worms were scored for live, dead, or censored status every 1–2 days as described above.

### 4.9 Imaging and analysis: *C. elegans*

*Myo-3p::GFP* and *myo-3p::mitoGFP* worms were anesthetized with 100 mM levamisole hydrochloride and placed on a 2 % agarose pad on a glass slide covered by a coverslip. *myo-3p::GFP* worms were imaged with a Leica DMi8, and m*yo-3p::mitoGFP* worms with a Leica Stellaris 5X. For both microscopes, images were acquired with a 63× oil objective with a z-stack of 0.27/0.3 μM respectively. *Myo-3p::GFP* images were analysed using the MuscleMetrics plugin in FIJI [43]. Briefly, images were taken between the pharynx and vulva, and individual sarcomeres were analysed by creating a maximal projection and a threshold was set in FIJI. Images with a threshold were loaded into the MuscleMetrics plugin which analysed individual myosin fibres (complete methodology explained in original paper [43]). *Myo-3p::mitoGFP* images were loaded into Imaris (Oxford Instruments), where surfaces were reconstructed. In addition to Imaris, surfaces were reconstructed via the Mitochondria Analyzer plugin in FIJI. Briefly, images were taken between the pharynx and vulva and mitochondria in the individual sarcomeres were analysed in 3D. A threshold was set in FIJI on a 10–15 slide Z-stack and these images were loaded into the Mitochondria Analyzer 3D analysis (where analysis was performed on a per cell basis).

### 4.10 Data analysis and statistics

Data distribution was tested by a *Shapiro-Wilk* test and equality of variances was tested by a *Levene* test. According to the distribution, a *t*-test or *Wilcoxon* rank test was performed to compare groups. For metabolomics, a false discovery rate (FDR) of 0.5 and a *t*-test threshold of 0.1 was set. *C. elegans* lifespan data was analysed using a log-rank test or *Cox* proportional hazards model, data was right censored with a death event scored as 1, and lost or censored worms scored as 0. Power analyses were performed to predetermine sample sizes for *C. elegans* microscopy. All statistical analysis and graphics were performed in R.

## CRediT authorship contribution statement

**Steffi M. Jonk:** Writing – review & editing, Writing – original draft, Project administration, Methodology, Investigation, Formal analysis, Data curation. **Alan Nicol:** Formal analysis, Data curation. **Vicki Chrysostomou:** Methodology, Investigation. **Emma Lardner:** Investigation. **Shu-Che Yu:** Investigation. **Gustav Stålhammar:** Supervision, Methodology, Investigation. **Jonathan G. Crowston:** Writing – review & editing, Supervision, Resources, Methodology, Funding acquisition. **James R. Tribble:** Writing – review & editing, Supervision, Project administration, Investigation, Data curation. **Peter Swoboda:** Writing – review & editing, Writing – original draft, Supervision, Resources, Project administration, Methodology, Investigation, Funding acquisition, Formal analysis, Data curation. **Pete A. Williams:** Writing – review & editing, Writing – original draft, Supervision, Resources, Project administration, Investigation, Funding acquisition, Formal analysis, Conceptualization.

## Declaration of competing interest

None.

## Data Availability

All data generated is available within the manuscript. Additional raw, unprocessed data can be made available upon request.
