# Supplementary material for: Metabolic analysis of sarcopenic muscle identifies positive modulators of longevity and healthspan in C. elegans
Source: Redox Biol. 2025 Jun 14;85:103732. doi: 10.1016/j.redox.2025.103732 (PMC12226095; doi:10.1016/j.redox.2025.103732)
Supplement: Multimedia component 1 [file mmc1.docx]

**Metabolic analysis of sarcopenic muscle identifies positive modulators of longevity and healthspan in *C. elegans***

**Authors:** Steffi M Jonk^1^, Alan Nicol^1^, Vicki Chrysostomou^2^, Emma Lardner^1^, Shu-Che Yu^1^, Gustav Stålhammar^1^, Jonathan G Crowston^2,3^, James R Tribble^1^, Peter Swoboda^4^, Pete A Williams^1^*

**Affiliations:**

^1^Department of Clinical Neuroscience, Division of Eye and Vision, St. Erik Eye Hospital, Karolinska Institutet, Stockholm, Sweden,

^2^Centre for Vision Research Duke-NUS & Singapore National Eye Centre, Singapore,

^3^Save Sight Institute at the University of Sydney and Royal Prince Alfred Hospital, Sydney, Australia,

^4^Department of Medicine Huddinge (MedH), Biosciences and Nutrition Unit, NEO, Karolinska Institutet, Huddinge, Sweden.

*To whom correspondence should be addressed: Pete A Williams, Department of Clinical Neuroscience, Division of Eye and Vision, St. Erik Eye Hospital, Karolinska Institutet, Stockholm, Sweden. pete.williams@ki.se

**This file includes:**

Supplementary

Figure S1: Additional skeletal muscle: histology and metabolomics.

Figure S2: Additional *C. elegans*: longevity, WormLab and microscopy.

Figure S3:  Additional *C. elegans*: oxidative and genetic stress.

Table S1: Antibodies used.

Table S2: *C. elegans* strains used.

Table S3: Chemicals used in *C. elegans* assays.

Table S4: Summary results of flooding assay.

Table S5: Summary results of survival assay.

Table S6: Summary results of oxidative stress survival assay.

Table S7: Summary results of mutant lines survival assay.

Table S8: Summary results of mutant sod-1 *C. elegans* strains under oxidative stress.

**
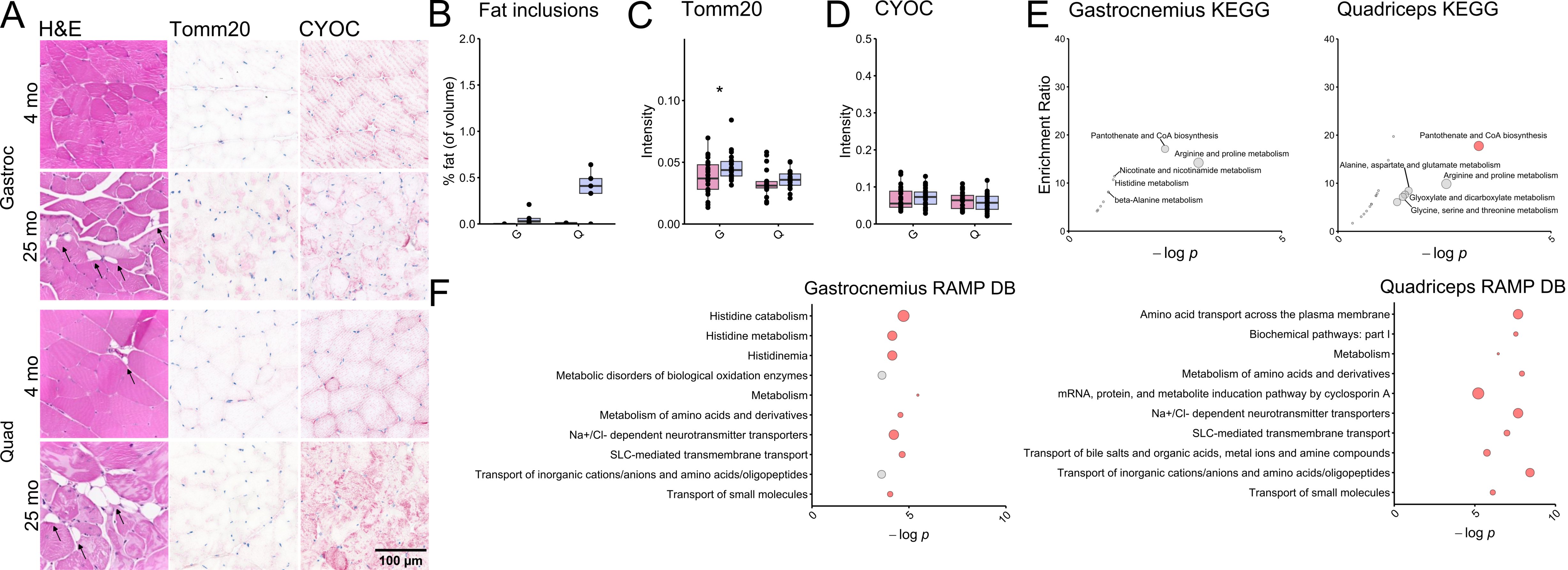
Figure. S1. Additional skeletal muscle: histology and metabolomics**. (**A-F**) C57BL/6J mice. (**A**) By histological assessment, we observed that (**B**) there was a slight increase of adipocyte infiltration during aging in the quadriceps muscle (ns), (**C**) Tomm20 intensity increased, but (**D**) CYOC intensity did not change. (**E**) A KEGG enrichment analysis shows the top 5 affected pathways in gastrocnemius and quadriceps, of which only pantothenate and CoA biosynthesis were significant. (**F**) Ramp DB enrichment shows the top 10 processes that significant hits are involved in. For KEGG and Ramp DB plots, red dots show an FDR<0.05, grey dots show an FDR>0.05 and dot size is based on the number of hits (between 1-3) and the enrichment ratio (between 3-13). *Pink* = 4 months of age, *purple* = 25 months of age. *p = * < 0.05, ** < 0.01, *** < 0.001, **** < 0.0001.*

**
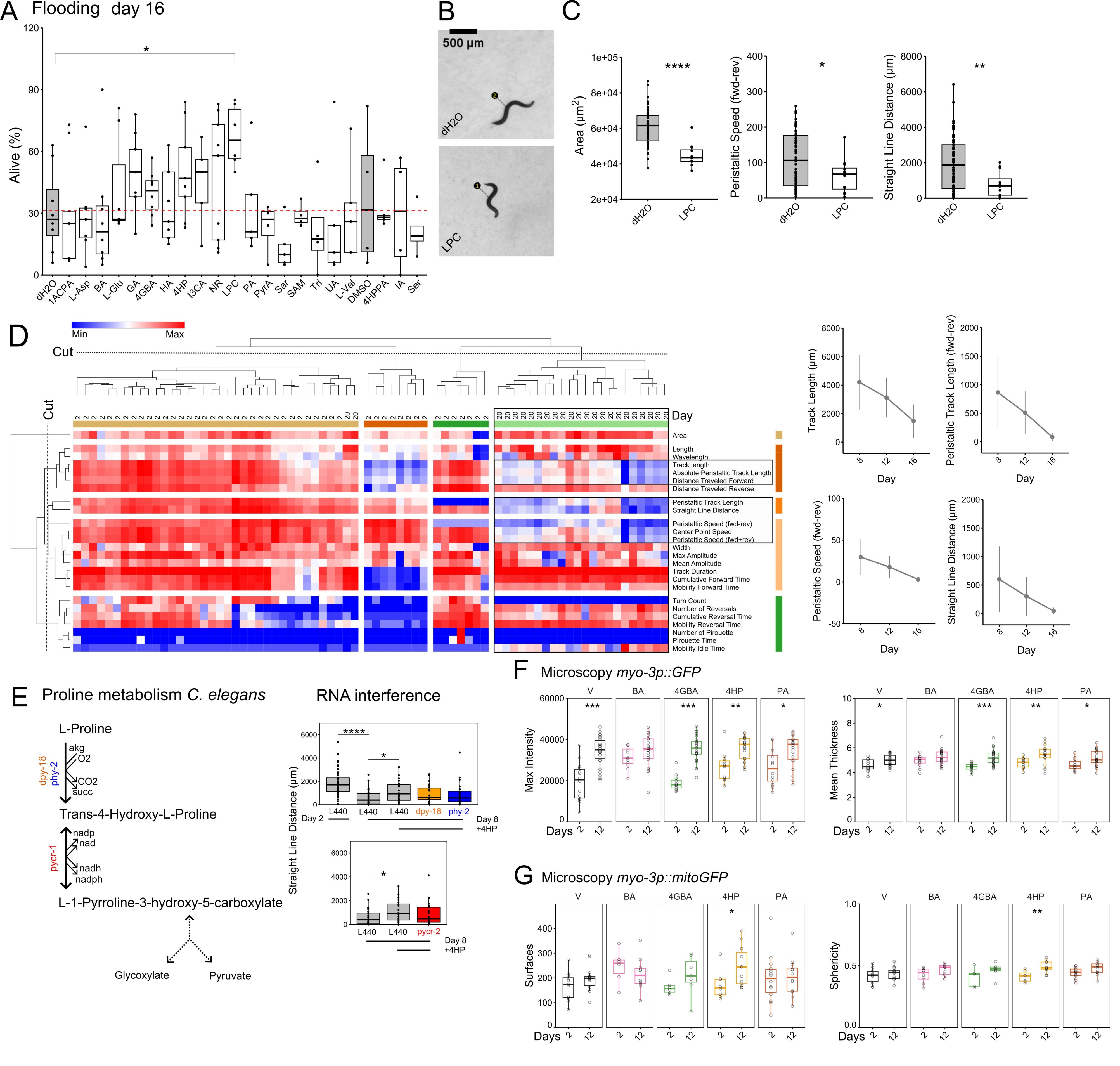
Figure. S2. Additional *C. elegans*: longevity, WormLab and microscopy.** (**A-E**) *C. elegans,* wild-type N2**,** (**F**) *C. elegans*, RW1596, (**G**) *C. elegans*, SJ4103 (cf. Suppl. Table S2). All metabolite candidates were supplemented at 5 mM. (**A**) Flooding assays identified potential healthspan enhancers after 5 mM supplementation from day 1, at day 16 of adulthood. Based on these results BA, 4HP, 4GBA and PA were selected for survival analysis. Note: Although LPC had the only significant effect in the flooding assay these worms were unhealthy from start of supplementation as recorded by images from the Wormlab (**B, C**): LPC treated worms were smaller and had decreased movement. (**D**) Using the Morpheus (Broad Institute) tool and with hierarchical clustering by Euclidian distance an aging locomotion phenotype was established were track length (center point trajectory), peristaltic track length (forward – reverse), peristaltic speed (forward – reverse) and straight-line distance showed an obvious decrease in the aged group (black box) decreased over time (selection of parameters are shown in the black boxes). (**E**) A representation of the proline pathway in *C. elegans* adapted from Wormflux.umassmed.edu. We downregulated the genes in orange, blue and red via RNA interference. Straight-line distance at day 8 of adulthood showed rescued movement with 4-hydroxyproline but not when RNA-interfered with *dpy-18, phy-2 and pycr-1*. (**F**) Fluorescence microscopy images of *myo-3p::GFP* (strain RW1596) were quantified, where an increase of max intensity is identified due to the intensity of the blebs, the mean thickness increased. BA did not show any significant effects, suggesting a protective effect on myosin during aging. (**G**) Fluorescence confocal microscopy images of *myo-3p::mitoGFP* (strain SJ4103) were quantified, where a similar number of mitochondrial surfaces was identified as well as a similar sphericity.1ACPA: 1-aminocyclopropanecarboxylic acid; L-Asp: L-asparagine; BA: beta-alanine; L-Glu: L-glutamine; GA: glyceric acid; 4GBA: 4-guanidinobutanoic acid; HA: hippuric acid; 4HP: 4-hydroxyproline; I3CA: indole-3-carboxylic-acid; NR: nicotinamide riboside; LPC: L-palmitoyl carnitine; PA: pantothenic acid; PyrA: pyroglutamic acid; Sar: sarcosine; SAM: S-Adenosyl methionine; Tri: trigonelline; UA: urocanic acid; L-Val: L-valine; 4HPPA: 4-hydroxyphenylpyruvic acid; IA: Indoleacetic acid (auxin); Ser: serotonin.. *p = * < 0.05, ** < 0.01, *** < 0.001, **** < 0.0001.*

**
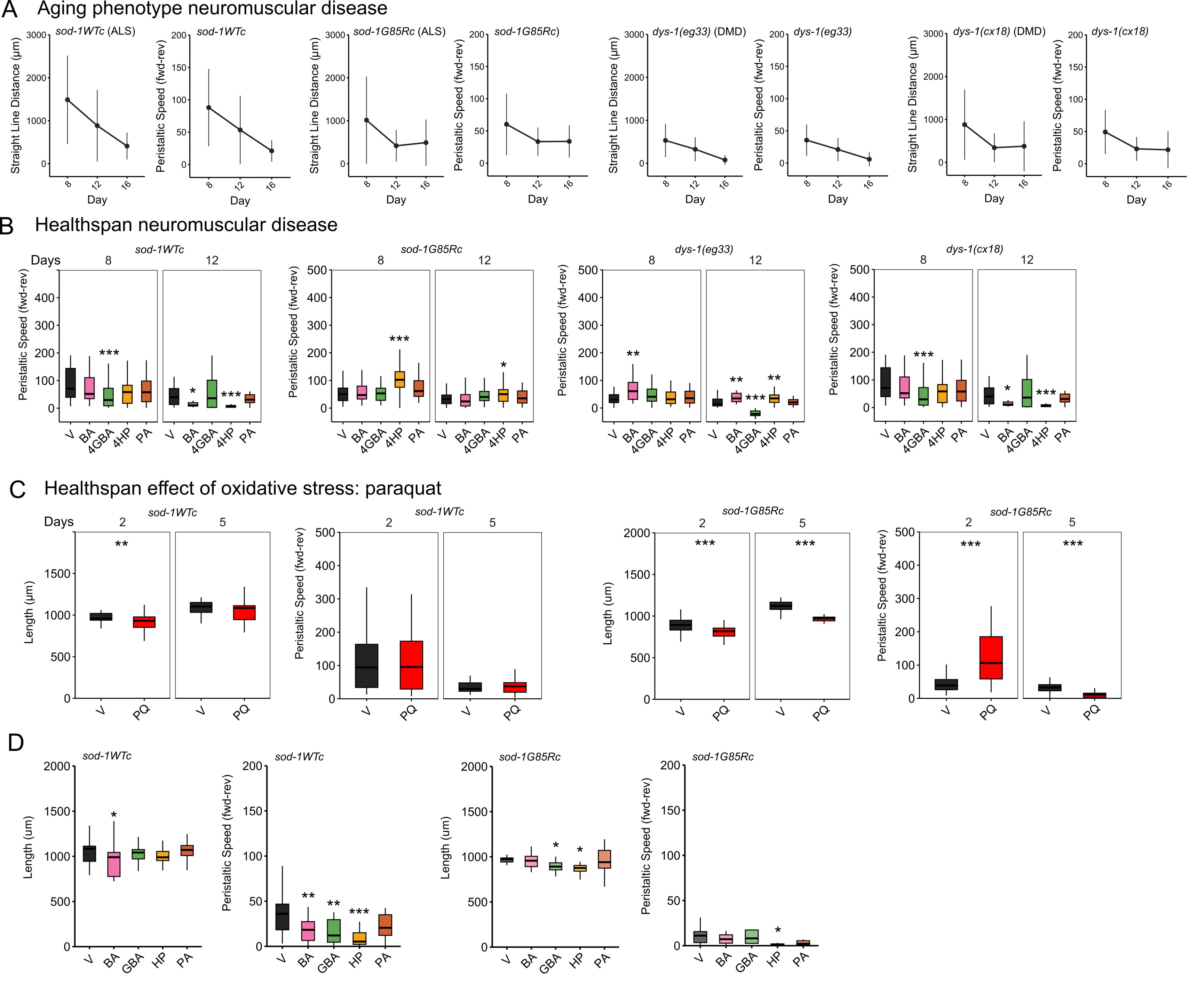
Figure. S3. Additional *C. elegans:* oxidative and genetic stress.** *C. elegans* strains: (**A**) HA2986 (*rt448*), HA3299 (*rt451*), BZ33 (*eg33*), LS292 (*cx18*), (**B-D**) (*rt448*), (*rt451*)**.** All metabolite candidates were supplemented at 5 mM. **(A**) An aging locomotion phenotype by decreased straight-line distance and peristaltic speed (forward – reversal) was observed in the sod-1 WT^C^, G85R^C^, dys-1(eg33) and dys-1(cx18) neuromuscular strains. (**B**) Healthspan analysis by locomotion (peristaltic speed (fwd-rev)) identified 4HP as a healthspan modulator in the ALS sod-1 G85R^C^ model, but not in the WT^C^ model and BA as a healthspan modulator in the DMD model (*eg33*). (**C**) Healthspan analysis by phenotype (length) and locomotion (peristaltic speed (fwd-rev)) at day 5 of adulthood demonstrated decreased size and locomotion for paraquat treated sod-1 G85R^C^. (**D**) Healthspan analysis by phenotype (worm length) and locomotion (peristaltic speed) at day 5 of adulthood demonstrated decreased locomotion for paraquat + BA, 4GBA and 4HP treated WT^C^ and G85R^C^, while none of the metabolite candidates saved paraquat (16 mM, re-supplemented every 2 days) induced decreased locomotion. V: vehicle, BA: β-alanine, 4GBA: 4-guanidinobutanoic acid, 4HP: 4-hydroxyproline, PA: pantothenic acid*. p = * < 0.05, ** < 0.01, *** < 0.001, **** < 0.0001.*

**Supplementary Table 1.** Antibodies used.

| Target | Clone | # | Supplier | Dilution |
| --- | --- | --- | --- | --- |
| CYOC | Rabbit | ab150422 | Abcam | 0.32 µg/µl (1:700) |
| MTCO1 | Mouse | ab14705 | Abcam | 1:200 |
| MYH7B | Rabbit | ab240173 | Abcam | 10.46 µg/µl (1:100) |
| Myosin | Mouse | ab37484 | Abcam | 0.81-0.99 µg/µl (1:1000) |
| SDHB | Mouse | ab14714 | Abcam | 1:100 |
| Tomm20 | Rabbit | ab78547 | Abcam | 0.9-1 µg/µl (1:1000) |
| 8-OHdG | Rabbit | bs-1278R | Bioss Biosciences | 0.01 µg/µl (1:100) |

**Supplementary Table 2.** List of *C. elegans* strains.

| Strain name | Genotype | Relevant details |
| --- | --- | --- |
| N2 | Wild type strain. |  |
| BZ33 | *dys-1(eg33) I.* | *dys-1*: dystrophin related  (*eg33*): substitution allele |
| HA2986 | *sod-1(rt448[sod-1WTc]) II.* | *sod-1*: superoxide dismutase  WTc: CRISPR/Cas9 with silent codon change |
| HA3299 | *sod-1(rt451[sod-1G85R]) II*. | *sod-1*: superoxide dismutase  G85R: CRISPR/Cas9 G85R missense mutation in *sod-1* |
| LS292 | *dys-1(cx18) I*. | *dys-1*: dystrophin related  (*cx18*): allele |
| SJ4103 | *zcIs14 [myo-3::GFP(mit)]* | *zcls14: [myo-3::gfp(mit)]* |
| RW1596 | *myo-3(st386) V; stEx30.* | *myo-3*: myosin heavy chain  *st386*: allele  *stEx30*: *[myo-3p::GFP::myo-3 + rol-6]* |

**Supplementary Table 3.** List of chemicals used in *C. elegans* assays.

| Chemical | Supplier | Catalog # |
| --- | --- | --- |
| Agar | Sigma Aldrich | 05039-500G |
| Bacto-peptone | Sigma Aldrich | P6838-500G |
| β-alanine | Sigma Aldrich | PHR1349-1G |
| Calcium Chloride | Sigma Aldrich | C1016-100G |
| L-Carnosine | Sigma Aldrich | C9625-5G |
| Cholesterol | Sigma Aldrich | C8667-5G |
| Dimethyl sulfoxide | Sigma Aldrich | D2438-5X10ML |
| Epigallocatechin gallate | Sigma Aldrich | PHR1333-1G |
| 5-Fluorodeoxyuridine | Sigma Aldrich | F0503-100MG |
| 4-Guanidinobutanoic acid | Sigma Aldrich | G6503-5G |
| Glycine | Sigma Aldrich | 3570-500GM |
| 4-Hydroxyphenylpyruvic acid | Sigma Aldrich | 114286-1G |
| 4-Hydroxyproline | Sigma Aldrich | PHR1939-500MG |
| KH2PO4 | Sigma Aldrich | P5655-1KG |
| K2HPO4 | Sigma Aldrich | P3786-1KG |
| Levamisole hydrochloride | Sigma Aldrich | 31742-250MG |
| Liquid broth | Gibco | 10855-001 |
| M9 | Sigma Aldrich | M6030-1KG |
| Nicotinamide | PanReac AppliChem | A0959,0250 |
| Pantothenic acid | Sigma Aldrich | P5155-100G |
| Phosphate buffered saline | Gibco | 70011-044 |
| Sarcosine | Sigma Aldrich | 131776-100G |
| Sodium chloride | Sigma Aldrich | S5886-500G |

**Supplementary Table 4.** Summary results of *C. elegans* flooding assays. 1-ACPA: 1-Aminocyclopropanecarboxylic acid; I3CA: Indole-3-carboxylic-acid; SAM: S-Adenosyl methionine; IA: Indoleacetic acid (auxin).

| Compound | Dose (mM) | Day 16 (% alive, ±) | n |
| --- | --- | --- | --- |
| dH2O |  | 31±20 | 143/8 |
| 1-ACPA | 5 | 30±25 | 147/6 |
| L-Asparagine | 5 | 29±21 | 141/7 |
| B-Alanine | 5 | 28±28 | 154/8 |
| L-Glutamine | 5 | 42±25 | 137/6 |
| Glyceric Acid | 5 | 47±20 | 154/7 |
| 4-Guanidinobutanoic Acid | 5 | 40±11 | 134/8 |
| Hippuric Acid | 5 | 35±20 | 132/7 |
| 4-Hydroxyproline | 5 | 51±22 | 143/8 |
| I3CA | 5 | 44±21 | 95/5 |
| Nicotinamide Riboside | 5 | 47±30 | 139/9 |
| L-Palmitoyl Carnosine | 5 | 68±15 | 84/6 |
| Pantothenic Acid | 5 | 33±25 | 108/4 |
| Pyroglutamic Acid | 5 | 23±13 | 87/4 |
| Sarcosine | 5 | 14±11 | 97/5 |
| SAM | 5 | 29±6 | 76/4 |
| Trigonelline | 5 | 23±23 | 71/4 |
| Urocanic Acid | 5 | 26±33 | 102/5 |
| L-Valine | 5 | 31±25 | 103/4 |
| DMSO |  | 38±35 | 74/4 |
| 4-Hydroxyphenylpyruvic Acid | 5 | 33±13 | 100/4 |
| IA | 5 | 30±28 | 68/4 |
| Serotonin | 5 | 21±12 | 75/3 |

**n = number of events (censored worms excl.)/number of experimental NGM plates*

**Supplementary Table 5**. Summary results of *C. elegans* survival assay.

| Compound | Dose  (mM) | n | Mean lifespan  (d) | Mean effect  (%) | Max lifespan  (d) | Max effect  (%) | p-value  (Log-rank) |
| --- | --- | --- | --- | --- | --- | --- | --- |
| Vehicle (ddH2O) | 2.5% | 95/3 | 13.7 | 0.0 | 22 | 0.0 |  |
| β-Alanine | 0.2 | 84/2 | 15.9 | 16.1 | 32 | 45.5 | 0.11 |
|  | 1 | 74/3 | 15.0 | 9.5 | 34 | 54.5 | 0.27 |
|  | 5 | 88/2 | 17.0 | 24.1 | 34 | 54.5 | <0.0001**** |
| 4-Guanidinobutanoic acid | 0.2 | 67/2 | 15.2 | 10.9 | 29 | 31.8 | 0.17 |
|  | 1 | 52/2 | 15.3 | 11.7 | 33 | 50.0 | 0.15 |
|  | 5 | 50/3 | 15.1 | 10.2 | 29 | 31.8 | 0.4 |
| 4-Hydroxyproline | 0.2 | 104/2 | 15.8 | 15.3 | 32 | 45.5 | 0.022* |
|  | 1 | 75/2 | 15.0 | 9.5 | 34 | 54.5 | 0.54 |
|  | 5 | 90/3 | 16.0 | 16.8 | 36 | 63.6 | 0.022* |
| Pantothenic acid | 0.2 | 84/2 | 15.3 | 11.7 | 30 | 36.4 | 0.35 |
|  | 1 | 59/2 | 14.8 | 8.0 | 32 | 45.5 | 0.57 |
|  | 5 | 56/2 | 16.7 | 21.9 | 34 | 54.5 | 0.00038*** |

**n = number of events (censored worms excl.)/number of independent experiments*

**p-values were calculated with a Log-Rank test for every condition against the vehicle condition (BA, Gly, 4GBA, 4HP, PA against ddH2O, and 4HPPA against DMSO)*

**p = * < 0.05, ** < 0.01, *** < 0.001, **** < 0.0001.*

**Supplementary Table 6.** Summary results of *C. elegans* oxidative stress survival assay.

| Stressor | Compound | Dose  (mM) | n | Mean lifespan  (d) | Mean effect  (%) | p-value  (Cox-regression) |
| --- | --- | --- | --- | --- | --- | --- |
| Vehicle (ddH2O) |  | 2.5% | 15/2 | 17.1 |  |  |
| Paraquat | Vehicle (ddH2O) | 2.5% | 27/2 | 20.6 | 0.0 | 0.0212* |
|  | β-Alanine | 5 | 24/2 | 19.6 | -4.9 | 0.2761 |
|  | 4-Guanidinobutanoic acid | 5 | 35/2 | 19.9 | -3.4 | 0.5713 |
|  | 4-Hydroxyproline | 5 | 28/2 | 21.0 | 1.9 | 0.8684 |
|  | Pantothenic acid | 5 | 26/2 | 23.1 | 12.1 | 0.1449 |
| Vehicle (DMSO) |  | 2.5% | 15/2 | 17.1 |  |  |
| Rotenone | Vehicle (ddH2O) | 2.5% | 33/2 | 13.4 | 0.0 | 0.0376* |
|  | β-Alanine | 5 | 34/2 | 15.6 | 10.7 | 0.2291 |
|  | 4-Guanidinobutanoic acid | 5 | 25/2 | 18.0 | 22.3 | 0.2444 |
|  | 4-Hydroxyproline | 5 | 31/2 | 16.9 | 17.0 | 0.1644 |
|  | Pantothenic acid | 5 | 29/2 | 13.7 | 1.5 | 0.8657 |

**n = number of events (censored worms excl.)/number of independent experiments*

**p-values were calculated with a Cox-regression test for vehicle condition against stressor+vehicle and stressors+compound*

**p = * < 0.05, ** < 0.01, *** < 0.001, **** < 0.0001.*

*.*

**Supplementary Table 7.** Summary results of mutant *C. elegans* strains survival assay.

| Mutant strain  (genotype) | Compound | Dose  (mM) | n | Mean lifespan  (d) | Mean effect  (%) | p-value  (Cox-regression) |
| --- | --- | --- | --- | --- | --- | --- |
| HA2986 | Vehicle (ddH2O) | 2.5% | 33/1 | 20.2 | 0.0 |  |
| (*sod-1(rt448[sod-1WTc]) II*) | β-Alanine | 5 | 51/1 | 22.7 | 12.4 | 0.0787 |
|  | 4-Guanidinobutanoic acid | 5 | 42/1 | 23.4 | 15.8 | 0.1174 |
|  | 4-Hydroxyproline | 5 | 8/1 | 22.0 | 8.9 | 0.8740 |
|  | Pantothenic acid | 5 | 24/1 | 22.7 | 12.4 | 0.5500 |
| HA3299 | Vehicle (ddH2O) | 2.5% | 73/3 | 22.9 | 0.0 |  |
| (*sod-1(rt451[sod-1G85R]) II*) | β-Alanine | 5 | 67/3 | 22.5 | -1.7 | 0.9377 |
|  | 4-Guanidinobutanoic acid | 5 | 85/3 | 22.1 | -3.5 | 0.6058 |
|  | 4-Hydroxyproline | 5 | 73/3 | 22.5 | -1.7 | 0.6971 |
|  | Pantothenic acid | 5 | 85/3 | 20.7 | -9.6 | 0.0336* |
| BZ33 | Vehicle (ddH2O) | 2.5% | 68/3 | 29.4 | 0.0 |  |
| (*dys-1(eg33) I*) | β-Alanine | 5 | 109/3 | 29.8 | 1.4 | 0.118899 |
|  | 4-Guanidinobutanoic acid | 5 | 76/3 | 30.5 | 3.7 | 0.00650** |
|  | 4-Hydroxyproline | 5 | 115/3 | 28.8 | -2.0 | 0.96836 |
|  | Pantothenic acid | 5 | 77/2 | 26.3 | -10.5 | 0.00184** |
| LS292 | Vehicle (ddH2O) | 2.5% | 75/3 | 22.7 | 0.0 |  |
| (*dys-1(cx18) I*) | β-Alanine | 5 | 111/3 | 21.4 | -5.7 | 0.281233 |
|  | 4-Guanidinobutanoic acid | 5 | 101/3 | 19.1 | -15.9 | 0.000308*** |
|  | 4-Hydroxyproline | 5 | 90/3 | 20.8 | -8.4 | 0.052219 |
|  | Pantothenic acid | 5 | 69/3 | 20.0 | -11.9 | 0.017031* |

**n = number of events (censored worms excl.)/number of independent experiments*

**p-values were calculated with a Cox-regression test for vehicle condition against compound per genetic background*

**p = * < 0.05, ** < 0.01, *** < 0.001, **** < 0.0001.*

**Supplementary Table 8.** Summary results of mutant sod-1 *C. elegans* strains under oxidative stress.

| Mutant strain  (genotype) | Stressor | Compound | Dose  (mM) | n | Mean lifespan  (d) | p-value  (Cox-regression) |
| --- | --- | --- | --- | --- | --- | --- |
| HA2986 | Vehicle (ddH2O) | Vehicle (ddH2O) | 2.5% | 50/1 | 18.1 |  |
| (sod-1(rt448[sod-1WTc]) II) | Paraquat (20 mM) | Vehicle (ddH2O) | 5 | 41/1 | 10.7 | 1.37E-12**** |
|  |  | β-Alanine | 5 | 41/1 | 10.4 | 0.59615 |
|  |  | 4-Guanidinobutanoic acid | 5 | 39/1 | 9.5 | 0.04948* |
|  |  | 4-Hydroxyproline | 5 | 37/1 | 9.1 | 0.00838** |
|  |  | Pantothenic acid | 5 | 39/1 | 11.1 | 0.55291 |
| HA3299 | Vehicle (ddH2O) | Vehicle (ddH2O) | 2.5% | 96/1 | 20.7 |  |
| (sod-1(rt451[sod-1G85R]) II) | Paraquat (20 mM) | Vehicle (ddH2O) | 5 | 33/1 | 8.8 | <2E-16**** |
|  |  | β-Alanine | 5 | 25/1 | 8.7 | 0.61266 |
|  |  | 4-Guanidinobutanoic acid | 5 | 26/1 | 7.8 | 0.03191* |
|  |  | 4-Hydroxyproline | 5 | 23/1 | 7.33 | 0.00552** |
|  |  | Pantothenic acid | 5 | 29/1 | 8.7 | 0.96923 |

**n = number of events (censored worms excl.)/number of independent experiments*

**p-values were calculated with a Cox-regression test in vehicle condition for vehicle against paraquat and in paraquat condition for vehicle against compounds*

**p = * < 0.05, ** < 0.01, *** < 0.001, **** < 0.0001.*
